# Spiritual care by nurses in curative oncology: a mixed‐method study on patients’ perspectives and experiences

**DOI:** 10.1111/scs.12710

**Published:** 2019-05-16

**Authors:** Anne Ebenau, Marieke Groot, Anja Visser, Hanneke W. M. van Laarhoven, René van Leeuwen, Bert Garssen

**Affiliations:** ^1^ Helen Dowling Institute for Psycho‐oncological Care Bilthoven The Netherlands; ^2^ Department of Anesthesiology, Pain and Palliative Care Radboud University Medical Center Nijmegen The Netherlands; ^3^ Faculty of Theology and Religious Studies, Comparative Study of Religion University of Groningen Groningen The Netherlands; ^4^ University of Amsterdam (AMC/VUMC) Amsterdam The Netherlands; ^5^ Faculty of Health Care Christian University of Applied Sciences Viaa Zwolle The Netherlands; ^6^ Department Health Psychology Rijksuniversiteit Groningen Groningen The Netherlands

**Keywords:** nursing, patients’ experiences, mixed‐methods, spirituality, oncology, spiritual care

## Abstract

**Aims:**

Spirituality can be important in adjusting to the experience of cancer and its medical treatment. Since nurses have frequent contact with patients, they seem to have a unique role in providing spiritual care. Nurses consider spiritual care important; however, little is known about how patients in a curative setting experience and value spiritual care. Therefore, this study aimed to give insight into patients’ experiences with and opinions about spiritual care as provided by nurses in curative cancer care.

**Methods:**

This is a national, multicentre mixed‐methods study, combining a quantitative approach using questionnaires (n = 62) and a qualitative analysis of semi**‐**structured interviews (n = 61). Nonparametric tests were used for quantitative data, and qualitative data were analysed inductively.

**Findings:**

Most patients rarely received spiritual care by nurses. If spiritual care was provided, it mainly consisted of noticing problems and referring to other professionals. This appeared to be dependent on certain ‘triggers’, such as age. Structural discussions on spirituality with a nurse were experienced rarely. This was explained by, among other factors, the hospital setting. Yet, the majority (79%) of patients found the attention to spirituality sufficient or very good. Furthermore, a majority (58%) viewed spiritual care as a nursing task: nurses should notice spiritual problems and refer to other professionals, though extensively discussing patients’ spirituality was neither considered nurses’ task nor capability.

**Conclusions:**

Attention to spiritual care in a curative setting, though not so much desired by most patients, should be pursued, because of its importance in performing person**‐**centred nursing care and its positive impact on patients’ health. By training nurses in offering spiritual care in proactive and ‘nonactive’ (accepting) ways, spiritual care could be structurally offered in clinical practice in personalised forms. Since younger and less spiritual patients are not much satisfied with spiritual care by nurses, they need special attention.

## Introduction

Being diagnosed with cancer might challenge previous trust in life and could cause disruptions in how people view the world and their future. Therefore, attention to spiritual care (SC) is essential 4, 5. Nurses have a unique role in providing compassionate care for patients with cancer. They often engage in frequent and long**‐**term contact and are present at precarious moments 6. In general, they have more regular contact with patients than medical doctors, especially during the period of chemotherapy. This emphasises a responsibility for patients’ psychosocial, physical and spiritual well‐being.

SC by nurses has been extensively studied in palliative and end**‐**of**‐**life settings in oncological care. Discussing spirituality seems more accepted in these contexts than in other fields 7. However, less is known about SC by nurses in curative oncology and about patients’ perspectives on this topic. Spirituality, though, is a way to move beyond a medicalised model of illness and care, and can be applied in any care setting 8, 9. Therefore, studies on the role of spirituality in curative care are needed. Previous studies on this subject are most often from the USA. Since in Western Europe, people tend to be less religious than in the USA and perceptions are less religiously oriented, and more diverse and eclectic 10, 11, 12, 13, the findings of American studies should not be generalised to this context.

### Background

Nurses deem attention to SC important; however, they lack practical commitment to it 14, 15, 16, 17, 18. Lack of time and competency, and conceptual vagueness of spirituality are often mentioned as barriers to the actual provision of SC 9, 19, 20, 21, 22. Patients, however, are less unanimous in their opinions about the importance of SC provided by nurses. On the one hand, most patients seek spiritual support **–** if needed **–** from family, friends and religious community and do not perceive SC as central in the role of nurses 23, 24, 25. On the other hand, research shows that between 41% and 67% of patients with a variety of diagnoses would have liked attention to their spiritual concerns from healthcare professionals, including nurses. Although the literature shows varying percentages, it indicates that many patients (between 17% and 72%) do not, or minimally, receive SC 26, 27, 28, 29.

The limited attention to SC in clinical practice is worrisome since SC has been found to have positive effects on patients’ perceived quality of and satisfaction with care and has positive associations with well**‐**being. Patients, who experience more SC from hospital employees, report less depression, a better total quality of life (QoL), and more meaning and peace 9, 19, 26, 27, 28, 29.

The aim of the present study was to obtain insight into SC provided by nurses in curative oncology from the patients’ perspectives. Based on the results of our study, it is possible to foster daily practice, as well as inform the policy and education debate on this important topic. To understand the level of SC provided, the following quantitative questions are addressed in this study: (i) ‘How often do patients experience SC by nurses and which patient characteristics are associated with the patient's wish to receive more (or less) SC?’; (ii) ‘which patient characteristics are associated with receiving SC by nurses?’; and (iii) ‘do patients consider SC a nursing task and what patient characteristics are associated?’. To better understand the nature of patients’ views of SC, the following qualitative questions are examined as follows: (i) ‘In what way do patients experience SC?’; (ii) ‘is it the patients’ opinion that nurses should provide SC?’; (iii) ‘what are **–** in the patients’ views **–** hindering and stimulating factors for nurses to provide SC?’; and (iv) ‘which improvements in SC by nurses are suggested?’. Box 1 provides our definitions of spirituality and SC.

Box 1 **Definitions of spirituality and spiritual care**

Spirituality: the dynamic dimension of human life that relates to the way persons (individual and community) experience, express and/or seek meaning, purpose and transcendence, and the way they connect to the moment, to self, to others, to nature, to the significant and/or the sacred. The spiritual field is multidimensional: (i) existential challenges; (ii) value‐based considerations and attitudes; and (iii) religious considerations and foundations 1. A Dutch version of this definition was used 2.

Spiritual care: the attitude, attention and care (of a professional caregiver) that result in the patient's feeling of ‘being seen’, in which there is attention to life issues and in which (re)finding balance, resilience and inspiration are considered to be important 3.

## Methods

### Design

This study is a national, multicentre, convergent mixed**‐**method study using parallel data analysis 30, 31. We used descriptive statistics and determined association tests in the quantitative part. The qualitative component consisted of semi**‐**structured interviews which were analysed by coding techniques based on the grounded theory approach 32, 33, 34. Both methods are of equal importance. Although there are no official guidelines on reporting mixed**‐**methods studies, this article is guided by the use of GRAMMS (Good Reporting of a Mixed‐methods Study 35). Also, COREQ criteria (COnsolidated criteria for REporting Qualitative research) were used for the qualitative findings 36. An extensive description of the methodology of this study can be found in the study protocol 3.

### Sample

Eight hospitals were purposively chosen, based on their region (northern, mid or southern parts of the Netherlands) with each region representing a certain cultural and religious orientation, for example southern parts of the Netherlands being more Catholic 37. To represent different organisation types, hospitals were also selected on their size (small or big regional hospital, academic hospital).

We aimed to include sixty patients. At the end of their curative chemotherapy, they were approached with an information leaflet and recruited by oncology nurses. The nurses were instructed to obtain a sample that represents the variety of demographics and cancer types seen in curative cancer care. Patients were to fill in a reply card for participation (‘yes’, ‘no’ and ‘maybe’) and send it to the researchers. If they were interested, they were contacted by the researchers. In total, 43 potential respondents refused participation (reason not asked).

End of treatment was chosen, because at that moment, patients had had every opportunity of having made contact with nurses. Chemotherapy was chosen, because this type of treatment involves much contact with nurses. Further inclusion criteria were being (i) diagnosed with cancer (lung carcinoma excluded); (ii) able to speak and understand Dutch; (iii) 18 years and older; and (iv) medically treated with curative intent 3.

### Data collection

#### Questionnaires

All patients were asked for demographics and for completion of the European Organization for Research and Treatment of Cancer, Quality of Life Questionnaire‐C 30 version 3 (EORTC QLQ‐C30) and the Spiritual Attitude and Involvement List (SAIL) 38, 39, measuring patients’ QoL and attitudes and interest towards spirituality, respectively. See the protocol article for scales’ details 3.

#### Semi‐structured interviews

From June 2012 till March 2016, three experienced scientific researchers (MG, HK (see acknowledgements) and AE) held single and individual face**‐**to**‐**face interviews at the patients’ homes during which only the patient and the researcher were present. No prior relationship between the two existed.

An interview guide had previously been developed and tested with three patients. Interviews included the following topics: (i) illness trajectory; (ii) sources of strength and meaning; and (iii) opinions on spirituality and SC. Interviews lasted from 39 to 170 minutes. In total, 62 interviews were conducted. The audio recording of one interview failed, so it could not be used for analysis. After each interview, field notes were made about initial insights. If requested, patients received their interview transcripts.

#### Additional statements

Seven additional statements were presented to patients at the end of the interview, which were used to support the answering of the quantitative research questions. They covered the following topics: (i) being spiritual; (ii) being religious; (iii) spirituality playing a role in coping with disease; (iv) SC as a nursing task; (v) attention to spiritual topics by nurses; (vi) having had discussions with nurses about spirituality; and (vii) satisfaction with these conversations (Table 1).

**Table 1:** Demographic medical and psychological descriptives of the sample

		Possible range	Cronbach's alpha
Demographics			
Age in years (SD)	52,242 (10,551)	≥18	
Gender – no. female	76%		
No. higher education. incl. university	42%		
No. stable relation	77%		
Denomination			
None	61%		
Protestant	21%		
Catholic	15%		
Otherwise	3%		
Region			
North	26%		
Middle	39%		
South	36%		
Type of hospital			
Academic	26%		
Nonacademic (regional big and regional small)	74%		
Diagnosis			
Breast cancer	67%		
Colon cancer	12%		
Hodgkin and Non‐Hodgkin lymphoma	8%		
Other	13%		
Treatment			
Only cytotoxic chemotherapy	18%		
Combination therapy	82%		
Interview questions			
I consider myself spiritual		1–4	
Little or not	71%		
Somewhat	23%		
Very much	7%		
I consider myself religious		1–4	
Little or not	84%		
Somewhat	10%		
Very much	7%		
Spirituality plays a role in dealing with the disease and its treatment	2.410 (0.901)	1–4	
Little or not at all	54%		
Somewhat	34%		
Very much	12%		
I discussed spiritual themes with nurses	1.705 (0.937)	1–5	
Rarely or never	80%		
Sometimes	13%		
Regularly	7%		
Was that sufficient?			
No	13%		
Yes	87%		
In my opinion, spiritual care is a nursing task	3.500 (1.050)	1–5	
Disagree	25%		
I doubt	17%		
Agree or highly agree	58%		
In my experience, the attention for SC by nurses is…	2.088 (0.714)	1–3	
Insufficient	21%		
Sufficient	49%		
Very good	30%		
SAIL			
Higher scores: higher level of spirituality			
Meaningfulness	4.383 (0.605)	1–6	0.433
Trust	4.594 (0.538)	1–6	0.46
Acceptance	4.460 (0.594)	1–6	0.584
Caring for others	4.857 (0.623)	1–6	0.839
Connectedness with nature	4.533 (1.060)	1–6	0.870
Transcendent experiences	2.592 (0.931)	1–6	0.788
Spiritual activities	2.504 (1.028)	1–6	0.774
EORTC			
Higher scores: lower quality of life			
Physical functioning	1.617 (0.497)	1–4	0.719
Role functioning	2.317 (0.978)	1–4	0.922
Emotional functioning	1.582 (0.543)	1–4	0.780
Cognitive functioning	1.877 (0.810)	1–4	0.706
Social functioning	1.901 (0.836)	1–4	0.724
Fatigue	2.406 (0.846)	1–4	0.854
Pain	1.557 (0.748)	1–4	0.815
Global functioning	3.082 (1.330)	1–7	0.903

### Data analysis

#### Quantitative data

Quantitative analysis is based on 62 respondents. Due to this small number, we used nonparametric statistics (with SPSS‐22) that are rather insensitive to non‐normality of data and small sample size (often inherent to mixed‐methods study). Associations were determined between several predictors **–** demographic and medical variables and measures of patients’ spirituality **–** and the statements on spiritual nursing, as well as associations between the spiritual nursing statements and QoL.

The relationships between ordinal/interval variables were analysed with the Spearman correlation coefficient (SCC), between ordinal/interval variables and dichotomous variables with the Mann–Whitney U‐test (MWU), between ordinal/interval variables and nominal variables with more than two levels with the Kruskal–Wallis one‐way analysis of variance (KWA), and between nominal variables with the chi‐square test (Chi^2^) or the Fisher Exact test (FET). In all tests, the critical p‐value was 0.05 (two‐sided).

Because the numbers in some cells were rather small, a few characteristics were combined into categories (marital status, religion and type of hospital). A mean score of a subscale was not calculated if more than 50% of the responses to its items were missing. If 50% or less was missing, these scores were replaced by a mean score of the other items.

#### Qualitative data

After finishing the data collection, AE and MG read several interviews (which were transcribed by external professionals) to become familiar with the material. Thereafter, analysis was performed in several steps by the researchers, while using the mixed**‐**method analysis software package DEDOOSE version 6. First, to organise data, themes were derived from the research questions, which were then used to select text segments in five interview transcripts. With this deductive method, we aimed to structure the data, though not to determine them. The researchers kept an open view and recognised some overlap between the themes. The themes represented the highest abstraction level of the qualitative data 40, 41.

We used coding techniques based on the grounded theory approach during the next steps of inductive analysis 32, 33, 34, 40. First, open coding: codes that were composed of, or represented, the patients’ literal words were applied to the data 32, 33, 42. Second, axial coding: these codes were clustered into (sub)categories which belonged to a theme. A codebook was developed. Finally, selective coding: the codebook was used as a framework for analysing remaining interviews. We constantly compared the new interviews with the codebook, and if necessary we extended or adapted it 32, 33, 34, 43. The qualitative analysis was finalised after 34 interviews when data saturation was reached; no new examples of categories came up 44. All results are presented by research questions (i.e. themes) instead of presenting all categories separately.

### Validity and reliability/rigour

The use of different methodologies was chosen for the following reasons. First, the various research questions ask for a quantitative as well as a qualitative approach. Second, qualitative data may provide a better understanding of the quantitative findings. Vice versa, quantitative data can be interpreted more objectively and can provide an indication for the extent to which certain views are shared. Findings, thus, were aimed to complement each other (convergent design). Analysis of the different data sets was carried out separately (parallel), and their outcomes are compared, merged and weaved in the interpretation stage, that is in the Discussion section 30, 31.

Rigour was achieved in several ways. For the qualitative part, the interviewer postponed concrete questions about spirituality and SC until the end of the interview to avoid the possibility of a ‘quasi**‐**understanding’ and to stimulate the respondent to use her/his own words. During both deductive and inductive analyses, AE and MG agreed on all codes and categories and ambiguous text fragments were discussed 45. No member check was executed. For the quantitative part, we determined Cronbach's alpha for all subscales of both questionnaires, except for the symptom scales of the EORTC, as it is not logical to assume that symptoms of the same subscale necessarily co**‐**occur. Since, unexpectedly, the alphas of the first three subscales of the SAIL were unacceptably low, these have not been used in the present study 39. The alphas of the remaining SAIL and EORTC subscales were between 0.71 and 0.92.

## Results

### Patient characteristics

Sixty**‐**two patients were included. Their mean age was 53 years (Table 1). The majority of the sample was female, lived in a stable relationship, finished higher education, had been recruited in large regional hospitals and was not a member of any denomination. About two**‐**third of the patients were treated for breast cancer.

Only 39% considered themselves ‘somewhat’ or ‘very much’ spiritual and/or religious. For about half (54%) of the patients, spirituality did not play an important role in dealing with the disease, and in only a small group of patients (12%), it played ‘very much’ a role. Spirituality playing a role in coping with the disease was more often indicated by women (Table 2) and by patients who considered themselves spiritual and scored high on SAIL subscale ‘Spiritual activities’ (Table 3).

**Table 2:** Relationships between patients’ opinions about spiritual nursing care and demographic data

	Type of test	Statistical parameters	p‐value
Spirituality plays a role in dealing with the disease and its treatment			
Age	SCC	*r *= −0.072	0.583
Gender	MWU	|z| = −1.995	0.046
Education	SCC	*r *=* *0.109	0.402
Marital status	MWU	|z| = −1.763	0.078
Region	KWA	Chi^2^ = 0.430	0.806
Type of hospital	MWU	|z| = −0.372	0.710
I discussed spiritual themes with nurses			
Age	SCC	*r* = −0.011	0.932
Gender	MWU	|z| = −0.493	0.622
Education	SCC	*r* = −0.020	0.877
Marital status	MWU	|z| = −0.772	0.440
Region	KWA	Chi^2 ^= 1.365	0.505
Type of hospital	MWU	*r* = −0.929	0.353
Was that sufficient?			
Age	MWU	|z| = −2.039	0.041
Gender	FET		0.667
Education	MWU	|z| = −1.000	0.317
Marital status	FET		1.000
Region	FET		0.071
Type of hospital	FET		0.19
In my opinion, spiritual care is a nursing task			
Age	SCC	*r* = −0.048	0.715
Gender	MWU	|z| = −1.527	0.127
Education	SCC	*r *=* *−0.217	0.096
Marital status	MWU	|z| = −1.471	0.141
Region	KWA	Chi^2^ = 1.488	0.475
Type of hospital	MWU	|z| = −0.088	0.930
In my experience, the attention for spiritual care by nurses is sufficient			
Age	SCC	*r *=* *0.345	0.009
Gender	MWU	|z| = −1.159	0.246
Education	SCC	*r* = −0.024	0.858
Marital status	MWU	|z| = −0.517	0.605
Region	KWA	Chi^2^ = 1.090	0.580
Type of hospital	MWU	|z| = −0.148	0.883

**Table 3:** Relationships between patients’ opinions about spiritual nursing care and spiritual/religious characteristics of the patient

	Type of test	Statistical parameters	p‐value
Spirituality plays a role in dealing with the disease and its treatment (spiritual coping)			
Considers oneself spiritual	SCC	*r *=* *0.700	0.000
Considers oneself religious	SCC	*r *=* *0.228	0.077
I discussed spiritual themes with nurses	SCC	*r *=* *0.350	0.006
Was that sufficient?	MWU	|z| = −1.486	0.137
In my opinion, spiritual care is a nursing task	SCC	*r *=* *0.157	0.230
In my experience, the attention for spiritual care by nurses is sufficient	SCC	*r* = −0.133	0.323
SAIL – Caring for others	SCC	*r *=* *0.084	0.522
SAIL – Connectedness with nature	SCC	*r *=* *0.193	0.139
SAIL – Transcendent experiences	SCC	*r *=* *0.188	0.153
SAIL – Spiritual activities	SCC	*r *=* *0.413	0.001
I discussed spiritual themes with nurses			
Considers oneself spiritual	SCC	*r *=* *0.34	0.01
Considers oneself religious	SCC	*r *=* *0.190	0.142
Spiritual coping	SCC	*r *=* *0.350	0.006
Was that sufficient?	MWU	|z| = −0.237	0.812
In my opinion, spiritual care is a nursing task	SCC	*r *=* *0.268	0.038
In my experience, the attention for spiritual care by nurses is sufficient	SCC	*r *=* *0.31	0.02
SAIL – Caring for others	SCC	*r *=* *0.317	0.014
SAIL – Connectedness with nature	SCC	*r *=* *0.120	0.360
SAIL – Transcendent experiences	SCC	*r *=* *0.367	0.004
SAIL – Spiritual activities	SCC	*r *=* *0.337	0.009
Was that sufficient?			
Considers oneself spiritual	MWU	|z| = −2.683	0.007
Considers oneself religious	MWU	|z| = −1.160	0.246
Spiritual coping	MWU	|z| = −1.486	0.137
I discussed spiritual themes with nurses	MWU	|z| = −0.237	0.812
In my opinion, spiritual care is a nursing task	MWU	|z| = −1.888	0.059
In my experience, the attention for spiritual care by nurses is sufficient	MWU	|z| = −3.997	0.000
SAIL – Caring for others	MWU	|z| = −1.724	0.085
SAIL – Connectedness with nature	MWU	|z| = −0.339	0.735
SAIL – Transcendent experiences	MWU	|z| = −0.359	0.720
SAIL – Spiritual activities	MWU	|z| = −0.984	0.325
In my opinion, spiritual care is a nursing task			
Considers oneself spiritual	SCC	*r *=* *0.213	0.103
Considers oneself religious	SCC	*r *=* *0.021	0.875
Spiritual coping	SCC	*r *=* *0.157	0.230
I discussed spiritual themes with nurses	SCC	*r *=* *0.268	0.038
Was that sufficient?	MWU	|z| = −1.888	0.059
In my experience, the attention for spiritual care by nurses is sufficient	SCC	*r *=* *−0.011	0.936
SAIL – Caring for others	SCC	*r *=* *0.134	0.313
SAIL – Connectedness with nature	SCC	*r* = −0.045	0.734
SAIL – Transcendent experiences	SCC	*r *=* *0.084	0.529
SAIL – Spiritual activities	SCC	*r *=* *0.012	0.928
In my experience, the attention for spiritual care by nurses is sufficient			
Considers oneself spiritual	SCC	*r* = −0.150	0.264
Considers oneself religious	SCC	*r* = −0.020	0.885
piritual coping	SCC	*r* = −0.133	0.323
I discussed spiritual themes with nurses	SCC	*r *=* *0.305	0.021
Was that sufficient?	MWU	|z| = −3.997	0.000
In my opinion, spiritual care is a nursing task	SCC	*r* = −0.011	0.936
SAIL – Caring for others	SCC	*r *=* *0.011	0.934
SAIL – Connectedness with nature	SCC	*r *=* *0.271	0.043
SAIL – Transcendent experiences	SCC	*r *=* *0.169	0.217
SAIL – Spiritual activities	SCC	*r* = −0.088	0.524

### Quantitative findings

1a) How often do patients experience SC by nurses and what characteristics are associated with patient's wish to receive more (or less) SC?

The great majority (80%) of patients had rarely or never discussed spiritual themes with their nurses and considered spiritual discussions sufficient (87%). Patients judged SC sufficient or very good (79%) (Table 1). Table 2 shows that elderly people were more satisfied about their SC conversations, and said more often that SC by nurses was sufficient. Higher scores on the spirituality subscale ‘Connectedness with Nature’ was associated with considering SC sufficient (Table 3). Furthermore, patients who considered themselves spiritual scored higher on considering discussions on spiritual themes sufficient. Patients who had discussed spiritual themes, and found this sufficient, more often were also satisfied with SC in general. Patients, who found spiritual conversations insufficient, had worse overall QoL and emotional and physical functioning (Table 4).

**Table 4:** Relationships between patients’ opinions about spiritual nursing care and QoL

	Type of test	Statistical parameters	p‐value
Spirituality plays a role in dealing with the disease and its treatment (spiritual coping)			
Global QoL	SCC	*r *=* *0.167	0.202
Physical functioning	SCC	*r *=* *0.020	0.882
Role functioning	SCC	*r *=* *0.069	0.604
Emotional functioning	SCC	*r *=* *0.164	0.211
Cognitive functioning	SCC	*r *=* *0.058	0.662
Social functioning	SCC	*r* = −0.004	0.973
Fatigue	SCC	*r *=* *0.043	0.748
Pain	SCC	*r* = −0.154	0.239
I discussed spiritual themes with nurses			
Global QoL	SCC	*r *=* *0.035	0.791
Physical functioning	SCC	*r *=* *0.018	0.891
Role functioning	SCC	*r *=* *0.132	0.320
Emotional functioning	SCC	*r* = −0.033	0.802
Cognitive functioning	SCC	*r* = −0.091	0.488
Social functioning	SCC	*r* = −0.116	0.377
Fatigue	SCC	*r* = −0.076	0.568
Pain	SCC	*r* = −0.056	0.670
Was that sufficient?			
Global QoL	MWU	|z| = −3.028	0.002
Physical functioning	MWU	|z| = −2.092	0.036
Role functioning	MWU	|z| = −1.700	0.089
Emotional functioning	MWU	|z| = −3.182	0.001
Cognitive functioning	MWU	|z| = −0.790	0.430
Social functioning	MWU	|z| = −1.922	0.055
Fatigue	MWU	|z| = −1.938	0.053
Pain	MWU	|z| = −1.567	0.117
In my opinion, spiritual care is a nursing task			
Global QoL	SCC	*r *=* *0.350	0.007
Physical functioning	SCC	*r *=* *0.343	0.008
Role functioning	SCC	*r *=* *0.218	0.101
Emotional functioning	SCC	*r *=* *0.095	0.475
Cognitive functioning	SCC	*r *=* *0.167	0.206
Social functioning	SCC	*r *=* *0.152	0.251
Fatigue	SCC	*r *=* *0.260	0.049
Pain	SCC	*r *=* *0.144	0.276
In my experience, the attention for spiritual care by nurses is sufficient			
Global QoL	SCC	*r* = −0.208	0.124
Physical functioning	SCC	*r* = −0.039	0.776
Role functioning	SCC	*r *=* *0.021	0.878
Emotional functioning	SCC	*r* = −0.188	0.165
Cognitive functioning	SCC	*r* = −0.086	0.529
Social functioning	SCC	*r* = −0.074	0.588
Fatigue	SCC	*r *=* *0.078	0.570
Pain	SCC	*r *=* *0.022	0.875

1b) Which patient characteristics are associated with receiving SC by nurses?

Patients who considered themselves spiritual, considered spirituality important in coping with their disease, had high levels of spirituality (higher scores on three SAIL scales), considered SC to be a nursing task and found SC sufficient. They also had more often discussed spiritual themes with nurses (Table 3).

1c) Do patients consider SC a nursing task and what characteristics are associated?

A majority of the patients stated that SC is a nursing task and that nurses should provide SC (Table 1). Particularly patients, who had discussed spiritual themes more often, considered SC a nursing task (Table 3). Patients with lower physical functioning and global QoL, and a higher level of fatigue more often were of the opinion that SC is a nursing task (Table 4).

### Qualitative findings

2a) In what way do patients experience SC?

In the patients’ view, the majority of the nurses had genuine attention to the patient as a person. Nurses often ‘tuned in’ their behaviour to the individual by responding to patients’ wishes and desires. In the patient's perception, this attitude leads to a focus on patients’ social, emotional and physical aspects. In general, however, nurses did not have consistent attention to life issues and most patients did not bring up spiritual themes themselves. Open questions about patients’ spirituality were not a common experience. Nurses often asked how their patients were doing, but they seldomly asked questions like ‘What does this mean to you?’.

Furthermore, patients felt that nurses asked about sources of strength, mostly by showing interest in family, work and hobbies, which might, however, be different from spiritual sources of strength. Nurses seldomly reinforced the positive side of spirituality. The only exception was when nurses offered medical information and positive test results, by which they reassured patients in their hope for cure. This for some patients had more or less spiritual implications, namely a hope for recovery and wholeness and becoming the person they used to be.

In specific situations, nurses offered or referred to a psychologist or social worker, such as with negative outcomes of psychosocial assessment scales, an emotional patient or a specific patient's request. Humanistic, spiritual or religious counsellors were not often suggested or referred to.

Some patients stated that some nurses made subjective assumptions about patients’ health, based on various signals, such as patients’ old(er) age or patients having a small social network. Additionally, judgement on the basis of facial expressions and behaviour was mentioned. Some patients considered this practice a quality of empathising with a patient and appreciated it. Other patients, however, criticised it and pointed at the risk of taking things for granted.

‘Maybe nurses could ask more questions to their patients. Actually, they never ask something. They occasionally ask “how are you?”, but not “what do you consider pleasurable to do?” [..] Every person is different; every single person that undergoes chemotherapy is different, reacts differently [..] I realize that assessing is very hard, but they are free to ask’. ‐ P**‐**01**‐**42, female patient, 55 years old

2b) Is it the patients’ opinion that nurses should provide SC?

Only when explicitly asked by the interviewer, did patients start to talk about (the importance of) SC by nurses. They considered it modestly important and most often necessary for others, but not for themselves. Most patients did not consider it a nursing task to extensively go into spirituality with a patient. However, in any case, part of a nursing team should have at least some attention to this dimension. Especially, ‘noticing of and assessing potential spiritual problems’ and ‘offering and referring to other professionals’ with more substantive knowledge and suitable skills were mentioned as relevant tasks for SC.

Patient: ‘I think they [nurses] already have so many things to do. I think, they really try hard. They run around from one place to another. [..] “I think it [SC] is very important”, but do they have space for it? Is the number of nurses sufficient to perform all those tasks? Because it is an extra task’.‐ P**‐**05**‐**45, female, 47 years old

Many patients stated that they did not wish to talk about spiritual themes with nurses. They found spiritual support elsewhere, especially from family, friends, fellow sufferers or external (professional) caregivers. Further, for some patients, spiritual issues were just not a matter of attention at times of treatment. They focused on survival and treatment.

2c) What are – in the patients’ views – hindering and stimulating factors for nurses to provide SC?

Patients experienced that especially the oncological outpatient ward, where they received their chemotherapy, was not the most suitable and inviting setting to talk about existential issues. Several reasons came forward, such as beeping equipment, running nurses, a lack of privacy and a desire to keep up a positive atmosphere. In such a setting, SC is not the primary concern for nurses as well as for patients and is, therefore, considered an ‘extra’ task by some patients. Patients also refrained from asking questions, because of the perception that nurses did not have time for extensive conversations due to their workload and, thus, to give space to other patients ‘who needed it more’ than themselves. In this context, patients reconsidered their criteria of being satisfied with care: although nurses did have little time for extensive conversations, they did have the intention to sincerely care and did often try to come by for social talk.

‘I said “No, I can talk with my own family and my friends very well” [..] Meanwhile, she [the nurse] said “Yes, madam, alright, I suppose ‐ as that is the way you see it ‐ that if you have problems, you will let us know”. [..] The waiting room is crowded, so they could do without me’. ‐ P**‐**05**‐**07, female patient, 58 years old

Finally, brief encounters with different nurses prevented the building of a relationship based on trust and openness. Some patients though did not bother, because they had regular contact with a nurse practitioner, who **–** in general **–** had more time than the nurses on the ward. According to the patients, there was space for spiritual issues in these meetings, but actual conversations did not concern such issues.

Contrary to hindering factors, patients mentioned that the assertiveness and openness of patients and nurses, and a special relationship between a patient and a nurse **–** leading to trust **–** made them inclined to talk about nonmedical and, potentially, spiritual issues. Furthermore, patients told that they observed that nurses provided SC to other patients than themselves. These other patients often had few social contacts or were emotional, of old age, religious or in a palliative or terminal phase. These circumstances triggered nurses to provide spiritual or psychosocial care by providing privacy for conversations between nurse and patients and by referring to a spiritual caregiver.

d) Which improvements in SC by nurses are suggested by patients?

Only a few patients mentioned suggestions for improvements. These concerned a desire for more structural attention to the patient as a person by (i) taking more time and private space for getting to know a patient, if possible by the same nurse; (ii) going into the potential positive sides of having cancer; and (iii) asking open questions.

## Discussion

This mixed‐methods study was performed to develop an integral understanding of patients’ experiences with SC by nurses in curative oncology. According to the quantitative findings, 80% of the patients did not discuss spiritual themes with nurses and one‐fifth of the patients found attention for spiritual issues insufficient. Interviews as well as the literature show that attention for spirituality was hindered by the environment of the oncology department and nurses’ lack of time 21, 46. Furthermore, it is very likely that the inadequate training of nurses played also a role in the small amount of SC provided 16, 18, 20. Patients in this study stated that, if provided, SC mainly consisted of signalling problems and referring to other professionals. The supportive role of spirituality seemed under‐recognised, despite that patients from this study and other studies often considered spirituality a positive resource 47, 48, 49, 50, 51.

Qualitative findings show that nurses seemed to provide SC based on particular triggers, such as age, religion or bad health. A Swedish study too shows that nurses consider older patients, immigrants who actively practice religion, and those severely ill in special need of SC 22. Quantitative data showed that those who were more dissatisfied with SC by nurses were younger and less spiritual. Also, elderly people were more satisfied about their SC conversations. These results might indeed point at selective attention to patients’ spirituality. Congruently, patients from our study suggested improvements in person‐centred care and pointed at the risk of taking things for granted when nurses judged their patients. Nurses did not appear to ask open questions about spirituality much, which was also recognised in another study 52.

In response to the question whether SC is a nursing task, 17% of the patients expressed doubt and 25% disagreed. Qualitative findings showed that patients thought that SC was an ‘extra’ task. Patients indicated that, if necessary, they would find spiritual support elsewhere, which is confirmed by other studies 23, 24, 25, 51, 53. Patients from our study considered SC important, but mostly for other patients ‘who need it more’. They might refer to those with a poorer QoL, who indeed indicated more often that SC is a nursing task. Another reason that most patients may have felt that SC by nurses is less urgent, is because they considered themselves not spiritual or not religious (respectively 71 and 84%) and stated that spirituality did not or only minimally played a role in coping with their disease and treatment (54%). On the other hand, the patients with high levels of spirituality (high scores on three SAIL scales) considered SC to be a nursing task more often than those with low levels of spirituality.

The perspectives of patients in our study are in sharp contrast with the importance attached to SC by nurses themselves. Studies, though not Dutch, show that a great majority of nurses (between 83% and 96%) considers SC a fundamental aspect of nursing 14, 15, 16, whereas a small majority (58%) of the patients of this study finds SC a nursing task. Still, we would suggest that attention to SC by nurses in curative oncological care should be pursued, because it is important in performing patient**‐**centred care **–** as a counterbalance to the many technical aspects of their job **–** and the potential benefits of SC on QoL 9, 19, 26, 27, 28, 29, 54.

### Relevance to clinical practice

Nurses appeared to behave predominantly in a reactive mode: responding to the problems they observed, mostly by referring to other healthcare professionals. However, this reactive mode comes with a risk. With the practice of observing, judging and responding to particular triggers, such as a patient's old age, religion or emotions, other cues might be overlooked. In practice, the spiritual health of a patient, who is doing seemingly well and does not ‘meet’ these triggers, might be taken for granted and the patient, thus, does not receive SC. Another Dutch study, too, found that nurses often miss cues on spirituality 52.

The literature shows that more SC training for nurses is needed. Such education could improve nurses’ sensitivity to spirituality and enhance the integration of SC within clinical practice and patient's health and satisfaction with care 52, 55, 56, 57, 58, 59. We believe that attention to the patients’ spirituality by nurses could be made more consistent by training nurses in using both proactive and ‘nonactive’ modes, in addition to the reactive mode. Proactive SC by nurses would include timely attention to and recognition of spirituality, both its strengths and its struggles. The structural presentation of standardised, but open**‐**ended questions about spirituality, could be used as starting points to explore patient's spirituality.

Another approach, which was not mentioned by patients, is ‘the presence approach’. Nurses in such a mode do not behave actively; they rather listen and have open, accepting and alert attitudes. This nonactive mode is the opposite of nurses’ main tendency to react and take away problems, that is to being task‐oriented 60. Both modes (proactive and nonactive) would be important additions to nurses’ predominant reactive mode and would decrease the risk of missing cues and increase the attention of a nurse for the patient as a person.

### Future research

To find out which importance exactly is attached to SC by patients, they could be asked to prioritise nursing tasks. Furthermore, an intervention study with structural attention to every patient's spirituality could provide more insight into whether patients were more unsatisfied with SC as a consequence of poor health (as this study shows: worse overall QoL and emotional and physical functioning) or with a more critical attitude with younger age, or whether structural screening would indeed increase their satisfaction with SC.

### Strengths and limitations

This study has several strengths, namely: (i) the unique focus on curative oncological care from patients’ perspectives; (ii) the large sample size for a qualitative study; (iii) the use of both qualitative and quantitative research methods, by which a rather rich picture of SC by nurses in curative oncology was obtained; and (iv) the careful operationalisation of the concept of spirituality during interviews. Spirituality appeared for some patients to be a difficult concept and they were inclined towards a definition in terms of faith or religion, or towards vagueness. Also, SC was sometimes explained as a psychological intervention or as alternative medicine. These definitions are recognised in the literature 22, 61. While respecting patients’ individual definitions on spirituality 62 and asking them for clarifications during the interviews, the researchers also provided the projects’ definitions of SC and spirituality, so that data collection remained transparent and reliable.

A limitation was that the majority of patients were female and suffered from breast cancer. This is representative of the population of people with cancer 63, but may have obscured other perspectives. Furthermore, the number of 62 respondents for the quantitative analyses is rather small. However, as we selected patients in the three different regions of the Netherlands and in three types of hospitals, the sample has good representation.

## Conclusion

Most curative cancer patients had no or little interest in spirituality and in SC and were satisfied with the absence of, or the very limited, personal contacts with their nurses about spiritual themes. A majority, though, deemed SC a nursing task. In patients’ opinions, SC by nurses was mostly (to be) confined to noticing problems and referring to other disciplines for help. Also, it appeared dependent on certain ‘triggers’. Explicit and extensive conversations about spiritual themes were most often neither experienced nor desired and, if considered relevant, patients thought other patients might need it more. Despite these general findings, it is also true that one‐fifth of the patients considered SC insufficient. Of great help and in addition to nurses’ reactive practice, would be the training of nurses in proactive and ‘nonactive’ nursing practice. This would ensure SC for everyone.

## Conflict of interest

The author(s) declare that they have no conflict of interests.

## Author contributions

We declare that all authors (i) made substantial contributions to conception and design, or acquisition of data, or analysis and interpretation of data; (ii) were involved in drafting the manuscript or revising it critically for important intellectual content; and (iii) gave final approval of the submitted version.

## Ethical approval

The Ethical Review Committee of the University Medical Center Utrecht waived the need for ethical approval (the study did not fall within the remit of the Dutch Act on Human Research) 64. Patients were informed that participation was voluntary and they could withdraw at any time. All signed informed consent. Furthermore, patients’ burden was minimised by using two questionnaires only.

## Funding

This work was supported by the Dutch Cancer Society (KWF) / Alpe D'Huzes (grant number HDI 2010 ‐ 4871).

## Availability of data and materials

The data sets used and/or analysed during the current study are available from the corresponding author on reasonable request.
